# Isoliquiritigenin Impedes Breast Cancer Progression through PITX1–PFKP-Mediated Glycolysis Reprogramming

**DOI:** 10.32604/or.2026.077059

**Published:** 2026-04-22

**Authors:** Cong Liu, Zhenyu Zhang, Ronghua Feng, Mengsi Zeng, Hui Li, Mei Zhu, Lan Zhuang, Zongjuan Li, Tao Wu

**Affiliations:** 1 Changde Hospital, Xiangya School of Medicine, Central South University (The First People’s Hospital of Changde City), Changde, China; 2 University Hospital, Central South University, Changsha, China

**Keywords:** Breast cancer, PITX1, PFKP, metastasis, glycolysis, isoliquiritigenin

## Abstract

**Background:**

Breast cancer is the leading cause of cancer-related deaths in women, primarily due to distant metastasis. Metabolic reprogramming plays a critical role in tumor growth and spread, but the metabolic mechanisms underlying metastasis in breast cancer remain unclear. The primary objective of this study is to identify molecular targets mediating breast cancer progression and to evaluate whether targeting the metabolic reprogramming represents a potential therapeutic strategy.

**Methods:**

To uncover key metabolic regulators involved in breast cancer progression, we analyzed high-throughput RNA sequencing data and identified Paired Like Homeodomain 1 (PITX1) as a frequently upregulated oncogene. Its expression was further validated by immunohistochemistry, quantitative PCR, and western blotting across various metastatic breast cancer tissues. The correlation between PITX1 expression and patient survival was also evaluated. Functional assays were conducted to explore the role of PITX1 in promoting breast cancer proliferation and metastasis. As this study is primarily based on mechanistic cellular and bioinformatic analyses rather than clinical intervention trials, traditional clinical effect size metrics are not directly applicable. However, we have now ensured that all major findings include quantitative effect measurements (e.g., fold changes, hazard ratios where applicable, correlation coefficients) together with corresponding statistical significance values to improve clarity and transparency.

**Results:**

Elevated PITX1 expression was significantly associated with poorer overall survival, distant metastasis-free survival, relapse-free survival, and post-progression survival in breast cancer patients. Silencing PITX1 significantly reduced breast cancer cell proliferation and suppressed glycolysis. Mechanistically, we found that PITX1 transcriptionally activates Phosphofructokinase platelet (PFKP), a key glycolytic enzyme, thereby enhancing glycolytic flux to promote tumor growth and metastatic capacity. Notably, isoliquiritigenin was identified as a small-molecule inhibitor that targets the PITX1–PFKP axis, downregulating glycolysis and consequently suppressing breast cancer progression.

**Conclusion:**

Our findings uncover a novel oncogenic mechanism by which PITX1 promotes breast cancer progression and metastasis through glycolytic reprogramming. Targeting the PITX1–PFKP axis with isoliquiritigenin offers a promising therapeutic strategy for breast cancer treatment.

## 1 Introduction

Breast cancer (BC) remains the most commonly diagnosed malignancy among women worldwide and continues to represent the leading cause of cancer-related death in the female population [1]. According to a recent study, the five-year survival rate of primary BC patients after diagnosis is approximately 90% [2]. However, 33% of BC patients develop non-nodal distant metastases that reduce the 5-year overall survival rate to 23% [2]. Clinical evidence indicates that metastatic spread most frequently involves the lung, bone, liver, and brain [3,4]. For patients with breast cancer liver metastases (BCLM), systemic chemotherapy and endocrine therapies remain standard treatment options, extending median survival to roughly 18–24 months [5]. Nevertheless, substantial heterogeneity exists in therapeutic responsiveness, and many patients exhibit limited benefit from chemotherapy or targeted regimens [6,7]. The emergence of acquired resistance further compromises treatment efficacy in metastatic settings [8,9]. Consequently, elucidating the molecular drivers of breast cancer progression and identifying actionable therapeutic targets remain pressing priorities.

Isoliquiritigenin (ISL), a naturally occurring chalcone flavonoid isolated from licorice (Glycyrrhiza species), has recently attracted attention for its potential anti-cancer properties. Emerging evidence suggests that ISL interferes with multiple oncogenic signaling cascades, thereby suppressing tumor cell proliferation, invasion, angiogenesis, and metastatic dissemination across various malignancies. For example, Xie et al. showed that ISL suppresses breast cancer brain metastasis by downregulating circNAV3, thereby inhibiting tumor growth, invasion, angiogenesis, and blood–brain barrier penetration via the circNAV3/miR-4262/ST6GALNAC5/EGFR axis [10]. Another study revealed that ISL potentiated gemcitabine and 5-fluorouracil cytotoxicity in pancreatic cancer cells by targeting p38 MAPK, enhancing p38 phosphorylation, and suppressing p38-dependent autophagic flux, thereby increasing tumor cell death [11].

Multiple molecular pathways contributing to breast cancer invasion and metastasis have been described [12,13]. They applied the cancer stem cell (CSC) concept to the Hippo pathway in breast cancer, revealing a mechanistic basis for Hippo kinase regulation by cell polarity [13]. A distinct subset of metastasis-initiating cells derived from lung metastatic lesions has been reported to confer therapeutic resistance despite lacking intrinsic proliferative superiority [14]. In basal-like breast cancer, KLF5 promotes the proliferation, survival, and migration of cancer cells, which results in tumor growth [15]. Hou et al. reported that eHsp90α signaling to the LRP1-AKT axis promotes tumor lymphangiogenesis and lymph node metastasis in breast cancer [16]. The tumor microenvironment also plays a crucial role in metastatic progression. For instance, exosome-mediated miRNA transfer from cancer-associated fibroblasts can enhance migratory and invasive behavior through FAK signaling activation [17]. Despite these advances, the upstream molecular determinants that coordinate metabolic adaptation with metastatic potential in breast cancer remain insufficiently defined.

To better understand the molecular mechanisms underlying breast cancer progression, we performed integrative analyses of high-throughput RNA sequencing datasets to identify candidate transcriptional regulators potentially involved in tumor growth and metastasis. Among these candidates, Paired-Like Homeodomain Transcription Factor 1 (PITX1), a developmental homeobox transcription factor, emerged as a gene of interest due to its potential association with clinical outcomes. Although PITX1 has been implicated in tumorigenesis in several contexts, its role in cancer metabolic reprogramming, particularly glycolysis-associated pathways, remains insufficiently characterized. Moreover, whether lineage-related transcription factors such as PITX1 contribute to metastatic behavior through metabolic regulation has not been clearly defined. Based on these observations, this study aimed to investigate the functional role of PITX1 in breast cancer progression and to explore its potential involvement in glycolytic regulation. We further sought to determine whether PITX1 modulates the expression of the key glycolytic enzyme PFKP and whether targeting this axis may represent a potential therapeutic strategy. In addition, we examined whether isoliquiritigenin exerts anti-tumor effects through modulation of PITX1-dependent pathways.

## 2 Materials and Methods

### 2.1 Collection of Clinical Specimens

Fresh breast cancer tissues (*n* = 15), adjacent nonmalignant tissues (*n* = 15), and lung metastases (*n* = 8), liver metastases (*n* = 12), and brain metastases (*n* = 15) were acquired from the First People’s Hospital of Changde City and immediately placed into paraformaldehyde for fixation. Inclusion criteria: (1) Histopathologically confirmed primary breast carcinoma or metastatic lesions derived from breast cancer; (2) Availability of sufficient tissue for molecular analysis. Exclusion criteria: (1) Metastatic lesions with uncertain origin; (2) Inadequate tissue quality or quantity for analysis. The Clinical Medical Research Ethics Committee of the First People’s Hospital of Changde City approved this research (No. 2025-258-01), which was conducted in compliance with the Declaration of Helsinki. All breast cancer patients signed a written informed consent form before participating in this study.

### 2.2 Cell Culture and Reagent

All cell lines used in this research, including the normal mammary cell line MCF-10A and the breast cancer cell lines BT549, MDA-MB-231, SKBR3, BT474, MCF-7, and T47D, were commercially purchased from the ATCC (American Type Culture Collection; Manassas, VA, USA). All cell lines were maintained and cultured following the guidance and instructions of the ATCC library. In detail, all cells were cultured in DMEM (Gibco, Grand Island, NY, USA) or RPMI-1640 (Gibco) supplemented with 10% FBS and maintained in a humidified incubator (Thermo Fisher Scientific, MA, USA) at 37°C with 5% CO_2_. Before experiments, all cell lines were fingerprinted to ensure their validity and confirm the absence of mycoplasma contamination. Isoliquiritigenin (ISL) was purchased from MedChemExpress (MCE, Monmouth Junction, NJ, USA; Cat. No. HY-N0373). According to the manufacturer’s specifications, ISL purity is ≥98% (HPLC). The compound was dissolved in dimethyl sulfoxide (DMSO) to prepare a stock solution (e.g., 50 mM), aliquoted, and stored at −20°C protected from light. Working concentrations were freshly prepared by diluting the stock solution in culture medium immediately before use. Isoliquiritigenin is treated with breast cancer cells (MDA-MB-231, MCF-7, SKBR3) and non-tumorigenic breast epithelial cells (MCF-10A) in the concentration of 0, 10, and 20 μM in each experiment. The final DMSO concentration in culture medium did not exceed 0.1%. Vehicle control groups treated with equivalent concentrations of DMSO were included in all experiments. Treatment durations and concentrations are specified in each experimental subsection.

### 2.3 qRT-PCR Analysis

TRIzol reagent (Invitrogen, Carlsbad, CA, USA) was used to extract cellular RNA. RNA concentration and purity were assessed using a NanoDrop spectrophotometer (Thermo Fisher Scientific), and samples with A260/A280 ratios between 1.8 and 2.0 were used for further analysis. RNA integrity was verified by agarose gel electrophoresis. cDNA was synthesized using the PrimeScript RT Reagent Kit (Takara, Tokyo, Japan) with 1 μg of total RNA as input per reaction. SYBR Premix Ex Taq Kit by Takara (Tokyo) was used to conduct qRT-PCR. The reaction volume was 20 μL per well. Cycling conditions were as follows: initial denaturation at 95°C for 30 s, followed by 40 cycles of 95°C for 5 s and 60°C for 30 s. Melting curve analysis was performed to confirm amplification specificity. Each experiment included at least three independent biological replicates, and each sample was analyzed in triplicate (technical replicates). Relative gene expression levels were calculated using the 2^−ΔΔCt^ method, with β-actin serving as the internal control. The primers used for PITX1 amplification were F: 5^′^-CTAGAGGCCACGTTCCAGAG-3^′^ and R: 5^′^-TGGTTACGCTCGCGCTTAC-3^′^. The primers used for PFKP amplification were F: 5^′^-GCATGGGTATCTACGTGGGG-3^′^ and R: 5^′^-CTCTGCGATGTTTGAGCCTC-3^′^. The primers used for ACTB amplification were F: 5^′^-CATGTACGTTGCTATCCAGGC-3^′^ and R: 5^′^-CTCCTTAATGTCACGCACGAT-3^′^.

### 2.4 Western Blot Analysis

Total protein was extracted from the breast cancer cells with RIPA lysis buffer, and protein degradation was blocked with PMSF (Phenylmethylsulfonyl fluoride). Equal amounts of total protein (30 μg per lane) were mixed with 5× SDS loading buffer, denatured at 95°C for 5 min, separated by 10% SDS–PAGE, and electro-transferred onto PVDF membranes (0.45 μm pore size, Millipore, USA) at 300 mA for 90 min. Membranes were blocked with 5% non-fat dry milk dissolved in TBST (Tris-buffered saline containing 0.1% Tween-20) for 1 h at room temperature. After incubation with anti-PITX1 (1:2500; Abcam, Cambridge, MA, USA), anti-PFKP (1:1000; CST, Danvers, MA, USA), and anti-ACTB (1:3000; CST) primary antibodies at 4°C, the membrane was incubated with secondary antibodies (HRP-conjugated anti-mouse or anti-rabbit IgG, 1:5000; CST) for one hour at room temperature. Protein bands were visualized using enhanced chemiluminescence (ECL) reagent (Thermo Fisher Scientific) and imaged using a chemiluminescence detection system. All Western blot experiments were independently repeated at least three times (biological replicates). Band intensities were quantified using ImageJ software and normalized to β-actin.

### 2.5 Construction and Transfection of Plasmids

Full-length cDNAs of human PITX1 (Gencode Transcript: ENST00000265340.12, total 945 bp) were synthesized and cloned into the plasmid vectors pEZ-Lv201 for lentiviral stable transfection (GeneCopoeia, Rockville, MD, USA). PITX1 shRNA constructs and negative control shRNA (sh-NC) were synthesized by Shanghai GenePharma Co., Ltd. (Shanghai, China). Lentiviral transduction was performed following the manufacturer’s protocol. The shRNA sequences are now provided here (sh-PITX1: 5^′^-GCCUGCGGCUCAAGUCCAAGC-3^′^, sh-NC: 5^′^-CCTAAGGTTAAGTCGCCCTCG-3^′^). All transfections of the plasmids were conducted with the Lipofectamine 3000 kit (Invitrogen) following the manufacturer’s instructions.

### 2.6 Cell Counting Kit-8 (CCK-8) Assay

Cell viability was assessed using CCK-8 reagent (GK10001, GlpBio, Montclair, CA, USA) according to the manufacturer’s instructions. MDA-MB-231, MCF-7, and SKBR3 breast cancer cells (5 × 10^3^) were resuspended, seeded in 96-well plates at 37°C, and cultured for three days. CCK-8 solution was then added to each well of the 96-well plate and incubated for two hours at 37°C. A microplate reader (Multiskan FC Microplate Photometer, Thermo Fisher Scientific, Waltham, MA, USA) was used to measure the absorbance of each well at 450 nm. Background absorbance from wells containing medium and CCK-8 reagent without cells was subtracted from all readings. Each experiment included at least three independent biological replicates with three technical replicates per group.

### 2.7 Transwell Assay

Transwell migration assays were performed using 24-well Transwell chambers with 8-μm pore polycarbonate membrane inserts (Corning, USA). First, 5 × 10^5^ MDA-MB-231 breast cancer cells were resuspended and plated in the upper chambers of a Transwell plate (in medium without FBS), and a medium containing 20% FBS was added to the lower chambers. Cells were incubated for 24 h at 37°C. After incubation, non-migrated cells on the upper surface were gently removed using a cotton swab. Cells that migrated to the lower surface were fixed with 4% paraformaldehyde for 20 min and stained with 2.5% crystal violet for 15 min. The migrated breast cancer cells were imaged using the bright field model under a microscope (Nikon, Tokyo, Japan). Cells were counted manually in five randomly selected fields per insert at 200× magnification. Each experimental condition was performed in triplicate, and experiments were repeated independently at least three times.

### 2.8 Measurement of Glucose Consumption and Lactate Production

Glucose consumption and lactate production were measured using the Amplex Red Glucose/Glucose Oxidase Assay Kit (Invitrogen) according to the manufacturer’s instructions. Briefly, cells were seeded in 6-well plates and cultured under indicated experimental conditions. After 24 h, culture supernatants were collected and centrifuged to remove cell debris. Glucose concentration in the culture medium was measured, and glucose consumption was calculated by subtracting the remaining glucose level from that of fresh medium. Lactate levels were measured in the collected culture supernatants. All values were normalized to total cellular protein content, which was determined using the BCA Protein Assay Kit (Thermo Fisher Scientific). Data were expressed relative to control groups. Each experiment was performed with three independent biological replicates, each containing technical duplicates.

### 2.9 Promoter Luciferase Activity Assay

The promoter region of the PFKP gene (−2000 bp to the transcription start site) was amplified from human genomic DNA using PrimeSTAR Max DNA Polymerase (Takara), a high-fidelity hot-start DNA polymerase, according to the manufacturer’s instructions. The amplified fragments were cloned into the pGL3-Basic luciferase reporter vector (Promega, Madison, WI, USA) to generate the promoter reporter constructs. Predicted transcription factor binding sites within the PFKP promoter were mutated using a site-directed mutagenesis kit (EZmax Fast Mutagenesis Kit, Yeasen, Shanghai, China) following the manufacturer’s protocol. Briefly, mutagenic primers carrying the desired nucleotide substitutions were used for PCR amplification, and the parental plasmid was removed by Dpn I digestion prior to bacterial transformation. All constructs, including wild-type and mutant promoters, were confirmed by Sanger sequencing. P1: “ATAAGCCC” was mutated to “CGCCTAAA”; P2: “TTAATTCT” was mutated to “GGCCGGAG”; P3: “CTAACCCC” was mutated to “AGCCAAAA”; P4: “CTCATCCC” was mutated to “AGACGAAA”; P5: “TTAAGCTG” was mutated to “GGCCTAGT”.

The constructed reporter plasmids and the PFKP overexpression plasmid were cotransfected into cells for further experiments. After 48 h, the breast cancer cell lines MDA-MB-231 and MCF-7 were added to 96-well plates (3000 cells per well). Relative luciferase activity was measured using a luciferase enzyme reporter experiment system kit (Promega). Luminescence was measured using a Multiskan FC Microplate Photometer (Thermo Fisher Scientific).

### 2.10 Chromatin Immunoprecipitation (ChIP) Assay

Chromatin immunoprecipitation (ChIP) assays were performed to determine whether PITX1 directly binds to the promoter regions of PFKP in breast cancer cells. Briefly, MDA-MB-231 and MCF-7 cells were crosslinked with 1% formaldehyde for 10 min at room temperature to stabilize protein–DNA interactions. Crosslinking was quenched with 125 mM glycine for 5 min. Cells were washed with cold PBS and lysed using ChIP lysis buffer supplemented with protease inhibitors. Chromatin was sheared by sonication to obtain DNA fragments ranging from 200 to 500 bp. The lysates were centrifuged, and the supernatants containing sheared chromatin were collected. Equal amounts of chromatin were incubated overnight at 4°C with anti-PITX1 antibody (Abcam) or normal rabbit IgG as a negative control. Protein A/G magnetic beads (Thermo Fisher Scientific) were then added and incubated for 2 h at 4°C to capture antibody–chromatin complexes. After sequential washing, immunoprecipitated complexes were eluted, and crosslinks were reversed by incubation at 65°C overnight. DNA was purified using a DNA purification kit (Qiagen, Germany) according to the manufacturer’s instructions. Enrichment of specific PFKP promoter regions was quantified by qPCR using region-specific primers. Results were normalized to input DNA and presented as percentage of input. Each experiment was performed in three independent biological replicates.

### 2.11 Statistical Analysis

Statistical analyses for *in vitro* experiments were performed using SPSS 24.0 (IBM Corp., Armonk, NY, USA). Data are presented as mean ± standard deviation (SD). All experiments were conducted with at least three independent biological replicates. Differences between two groups were assessed using two-tailed Student’s *t*-test. A value of *p* < 0.05 was considered statistically significant. For bioinformatics analyses, publicly available RNA-sequencing data and corresponding clinical information for breast cancer were obtained from The Cancer Genome Atlas (TCGA-BRCA) database (https://portal.gdc.cancer.gov). Raw count data were downloaded and processed using R software (version 4.2.2). Data preprocessing and normalization were performed using the “DESeq2” package (version 1.38.3), which applies size factor estimation to normalize sequencing depth. Differential expression analysis was conducted using DESeq2, with genes considered significantly differentially expressed when |log_2_ fold change| ≥ 2 and false discovery rate (FDR) ≤ 0.05. Multiple testing correction was performed using the Benjamini–Hochberg method to control the false discovery rate. Correlation analysis between PITX1 and other genes was conducted using Pearson correlation coefficients in R. Genes with an absolute correlation coefficient ≥ 0.3 and *p* < 0.05 were selected for downstream analyses. Functional enrichment analyses, including Gene Ontology (GO) and Kyoto Encyclopedia of Genes and Genomes (KEGG) pathway analyses, were performed using the “clusterProfiler” package (version 4.6.2). Enrichment results were considered significant at adjusted *p* < 0.05. Survival analyses were performed using the “survival” (version 3.5-5) and “survminer” (version 0.4.9) R packages. Kaplan-Meier curves were generated using optimal cut-off values determined by the surv_cutpoint function. Hazard ratios (HRs) and corresponding 95% confidence intervals were calculated using Cox proportional hazards models. To investigate the biological role of PITX1 in breast cancer cells, we performed functional enrichment analysis of PITX1 using the Metascape algorithm (http://metascape.org). Expression data for PITX1 from the GEPIA database were retrieved using log_2_(TPM + 1) normalized expression values for visualization purposes. To explore potential transcriptional targets of PITX1, *in silico* motif prediction analysis was performed using the JASPAR database (JASPAR 2022 release, https://jaspar.genereg.net/). The position weight matrix (PWM) corresponding to the human PITX1 transcription factor was retrieved from the JASPAR CORE vertebrates collection. Promoter sequences (defined as −2000 bp to +100 bp relative to the transcription start site, TSS) of candidate genes were obtained from the UCSC Genome Browser (GRCh38/hg38 assembly). Motif scanning was conducted using the JASPAR web-based scanning tool with a relative profile score threshold of 80% to identify high-confidence PITX1 binding sites. Predicted binding sites with scores above the threshold were considered potential PITX1-responsive elements. Candidate target genes identified by motif prediction were further integrated with differential expression and correlation analyses to prioritize biologically relevant downstream genes. All bioinformatics analyses were conducted using R software unless otherwise specified. SPSS was used exclusively for statistical analyses of experimental (non-bioinformatic) data.

## 3 Results

### 3.1 PITX1 Is Upregulated and Associated with Worse Survival in Breast Cancer

**Figure 1:**
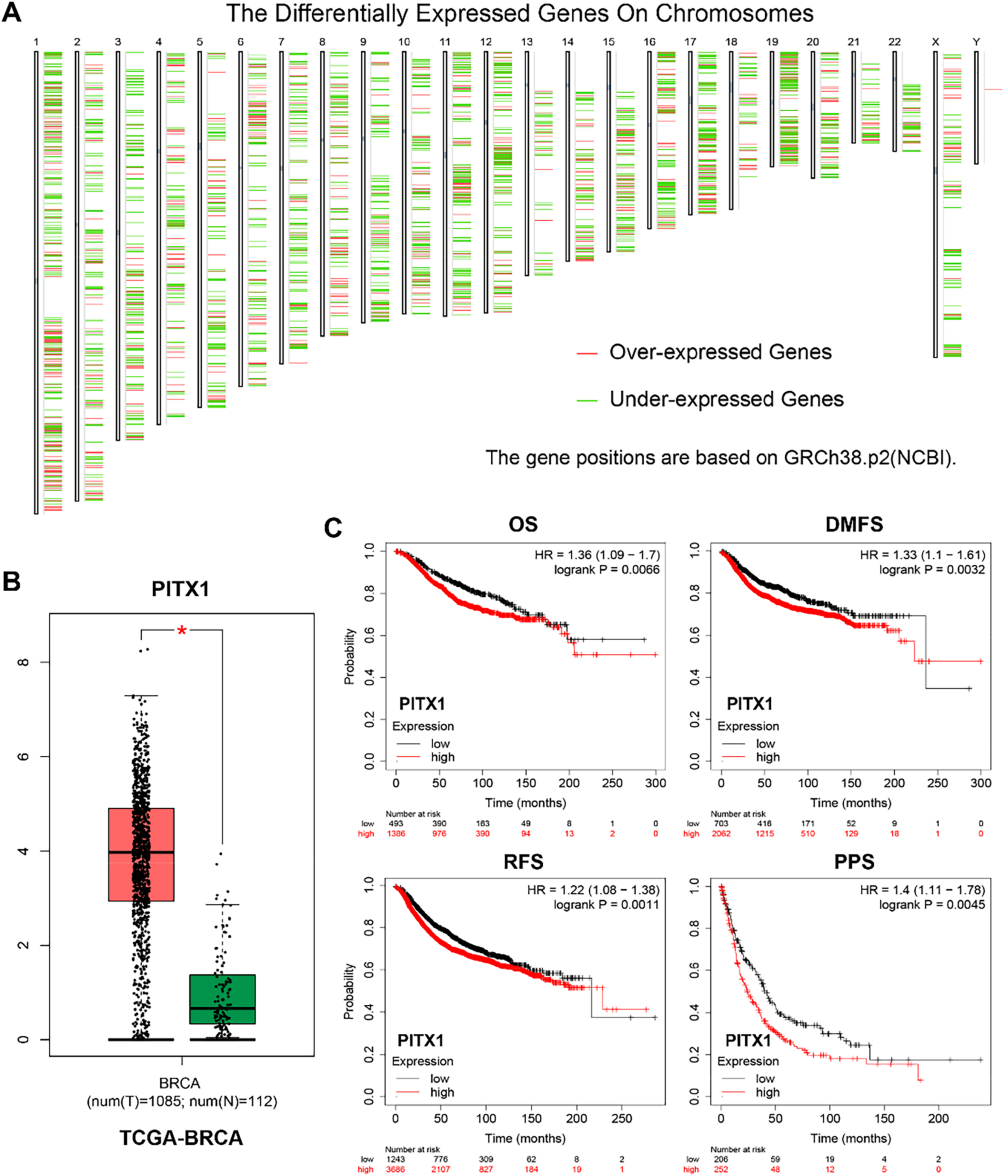
PITX1 is significantly upregulated in breast cancer. (**A**) The heatmap shows the differentially expressed genes on chromosomes between breast cancer and normal mammary tissues in the TCGA database. Red indicates upregulated genes, and green indicates downregulated genes. (**B**) The relative expression of PITX1 in breast cancer and normal mammary tissues was analyzed in the TCGA database. The data were obtained from the GEPIA public database using log_2_(TPM + 1) transformation for log-scale normalization. (**C**) Higher expression of PITX1 was associated with worse overall survival, distant metastasis-free survival, relapse-free survival, and post-progression survival in patients with breast cancer in KM Plotter cohorts. *p < 0.05.

**Figure A1:**
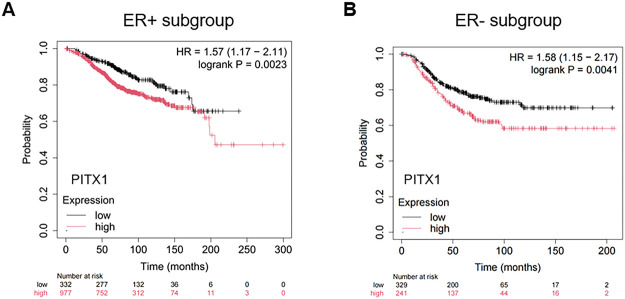

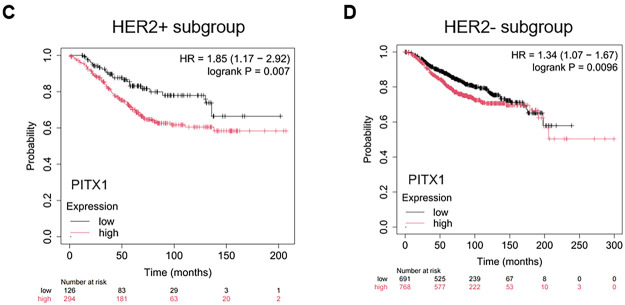
Prognostic significance of PITX1 expression in distinct molecular subtypes of breast cancer derived from the KM-plotter database. (**A**) ER^+^ group; (**B**) ER^−^ group; (**C**) HER2^+^ group; (**D**) HER2^−^ group.

To investigate the gene expression profile in breast cancer, we compared gene expression between breast cancer and normal mammary tissues in the TCGA database. The differentially expressed genes on chromosomes between breast cancer and normal mammary tissues in the TCGA database were shown (Fig. 1A). Differential gene expression analysis showed that PITX1 was significantly upregulated in breast cancer compared to normal mammary tissues (Fig. 1B). In addition, we found that higher expression of PITX1 was associated with worse overall survival, distant metastasis-free survival, relapse-free survival, and post-progression survival in patients with breast cancer in KM Plotter cohorts (Fig. 1C). We performed additional bioinformatic subgroup analyses stratified by molecular subtype. Notably, we found that high PITX1 expression was consistently associated with poorer overall survival in both ER-positive and ER-negative, as well as HER2-positive and HER2-negative breast cancer subgroups. These results strengthen the translational relevance of PITX1 as a prognostic factor across heterogeneous clinical contexts (Fig. A1).

**Figure 2:**
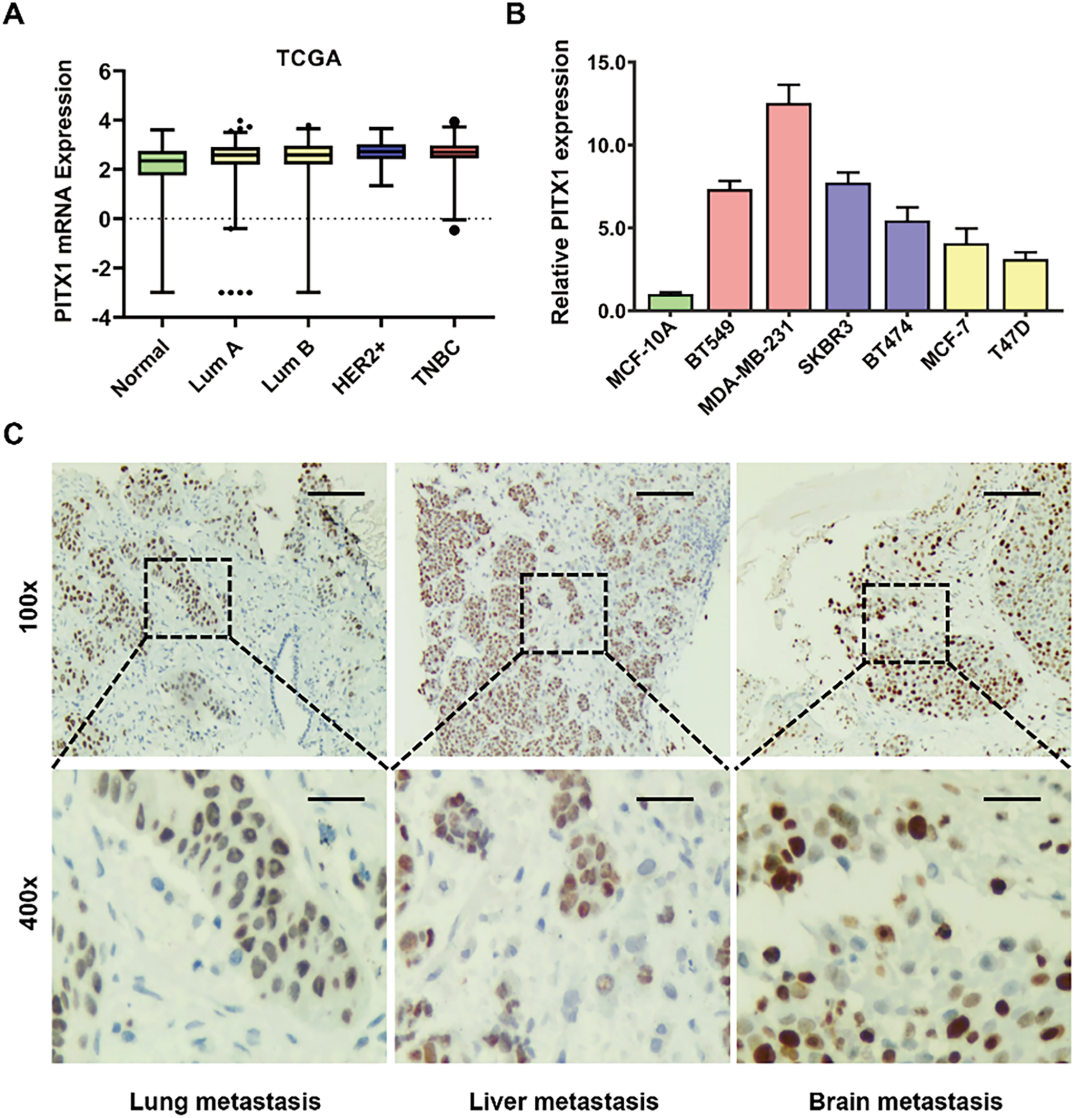
PITX1 is significantly upregulated in metastatic breast cancer. (**A**) The expression profile of PITX1 in different molecular subtypes of breast cancer in the TCGA database. (**B**) PITX1 was more highly expressed in breast cancer cell lines than in the normal breast epithelial cell line MCF-10A, as validated by qPCR analysis. (**C**) The PITX1 expression was evaluated by immunohistochemical staining in lung metastasis (*n* = 8), liver metastasis (*n* = 12), and brain metastasis (*n* = 15) of breast cancer. Representative images of immunohistochemical staining for PITX1 at different sites of breast cancer metastasis are shown. Scale bar: 200 μm (upper), 50 μm (lower).

**Figure A2:**
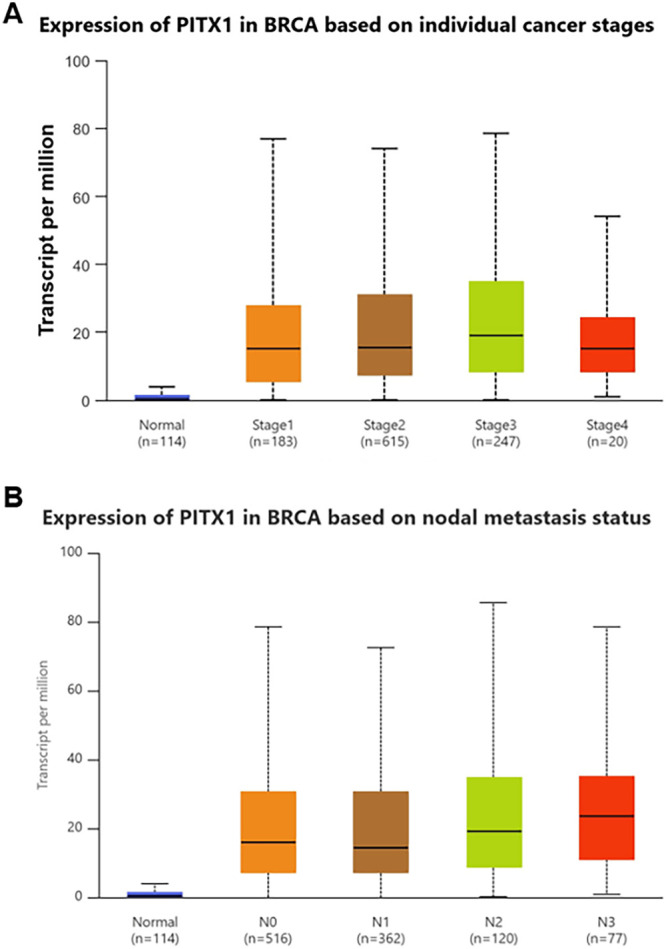
Expression of PITX1 in breast cancer patients across different TNM stages based on TCGA data. (**A**) Individual cancer clinical stages; (**B**) nodal metastasis status.

Next, we explored the expression profile of PITX1 in different molecular subtypes of breast cancer (Fig. 2A). We examined the expression level of PITX1 in various breast cancer cell lines by qPCR analysis. The six initial breast cancer cell lines were selected to represent major subtypes: luminal (MCF-7, T47D), HER2-positive (SKBR3, BT474), and triple-negative breast cancer (BT549, MDA-MB-231). This design allowed us to assess PITX1 expression patterns across molecular subtypes. The results revealed that PITX1 had the highest expression level in triple-negative breast cancer cell lines (BT549 and MDA-MB-231) (Fig. 2B). Additional bioinformatic analyses based on TCGA data have now been performed to examine PITX1 expression across different clinical stages, including clinical stage and TNM.N status (Fig. A2). To verify the expression level of PITX1 at different sites of breast cancer metastasis, we performed immunohistochemical staining. Our results showed that PITX1 had high expression in lung, liver, and brain metastases of breast cancer (Fig. 2C).

### 3.2 PITX1 Promotes the Proliferation and Increases the Metastatic Ability of Breast Cancer Cells

**Figure 3:**
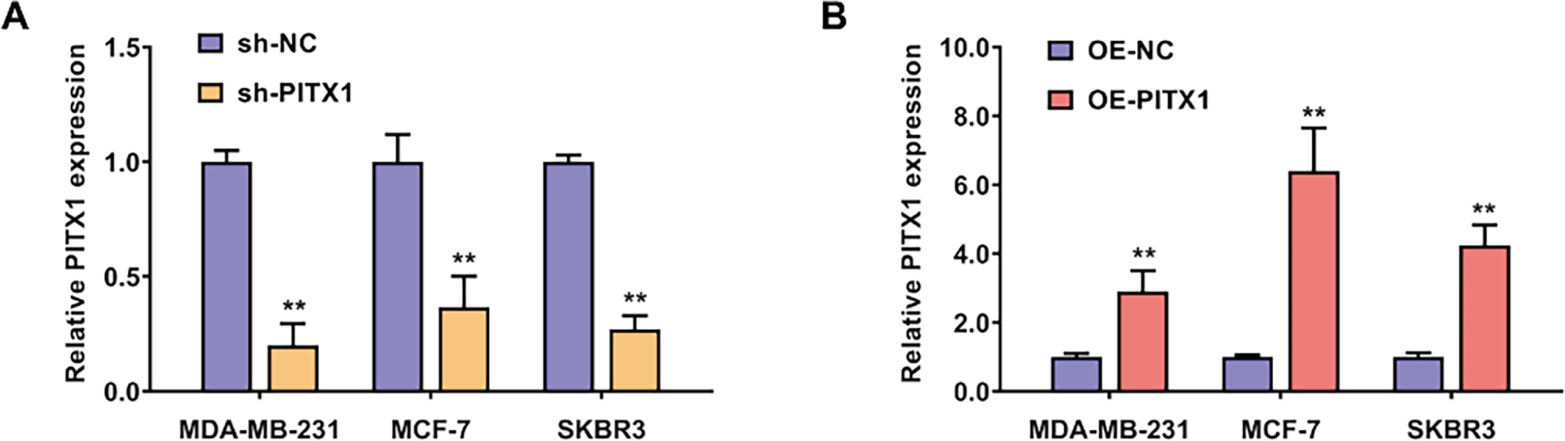

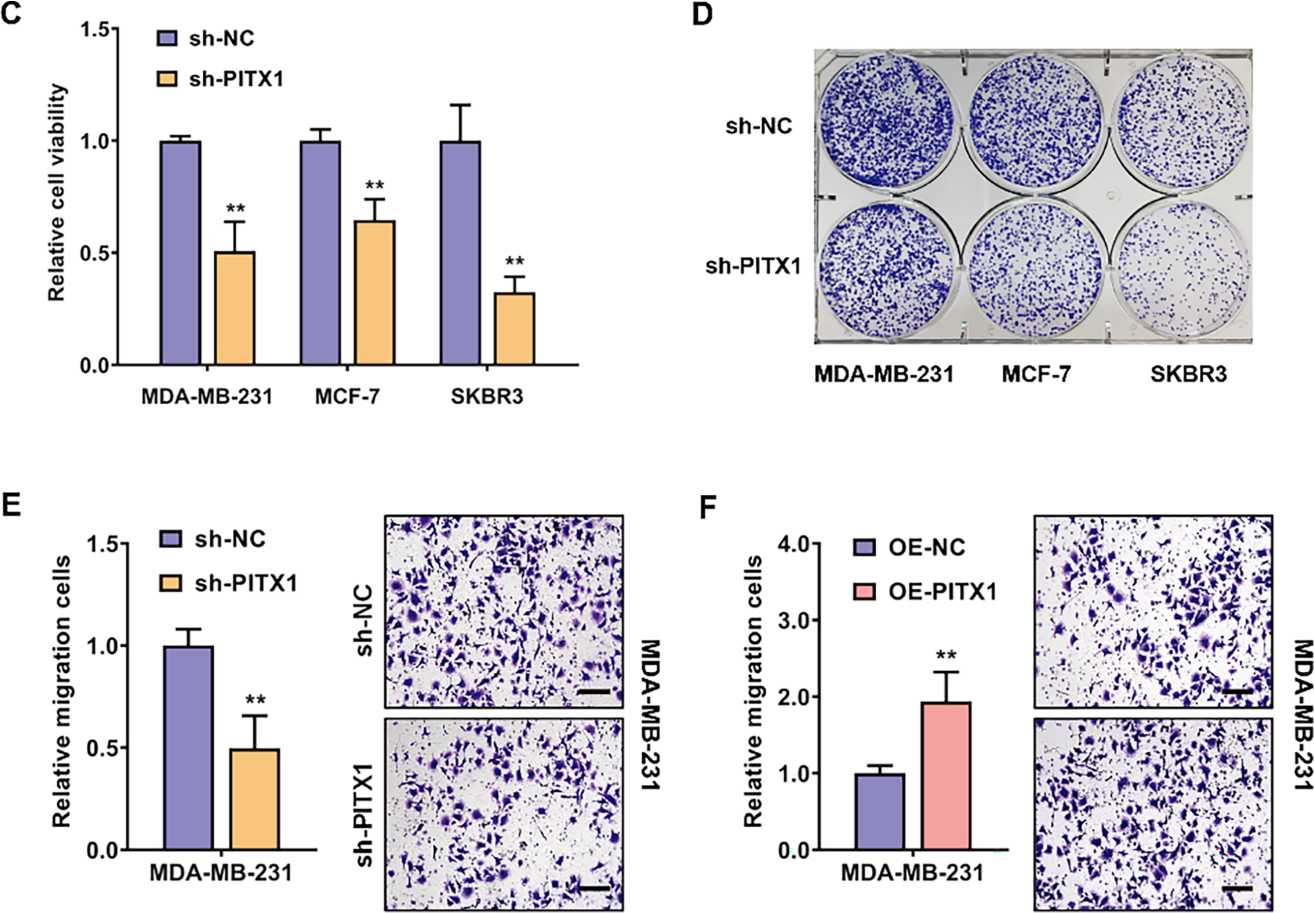
PITX1 promotes the proliferation and increases the metastatic ability of breast cancer cells. (**A**) The efficiency of shRNA knockdown of PITX1 was evaluated in MDA-MB-231, MCF-7, and SKBR3 breast cancer cells by qRT-PCR analysis. (**B**) The efficiency of exogenous overexpression of PITX1 was assessed in MDA-MB-231, MCF-7, and SKBR3 breast cancer cells by qRT-PCR analysis. (**C**) A CCK-8 assay was conducted to assess the growth rate of MDA-MB-231, MCF-7, and SKBR3 breast cancer cells. (**D**) A colony formation assay was performed in MDA-MB-231, MCF-7, and SKBR3 breast cancer cells. The representative images of three technical repeats are shown. At the same time, three biological repeats were also performed. (**E**) The Transwell assay showed that inhibition of PITX1 markedly decreased the migration ability of MDA-MB-231 breast cancer cells. (**F**) The Transwell assay showed that overexpression of PITX1 greatly increased the migration ability of MDA-MB-231 breast cancer cells. Scale bar: 50 μm. **p < 0.01.

Functional experiments were further conducted to investigate the potential functions of PITX1 in the progression and metastasis of breast cancer. We constructed MDA-MB-231 (triple-negative subtype), MCF-7 (luminal subtype), and SKBR3 (HER2-positive subtype) breast cancer cell lines with stable PITX1 overexpression or knockdown via lentiviral infection (Fig. 3A,B). Using qPCR analysis, we investigated the overexpression and knockdown efficiency in the breast cancer cell lines MDA-MB-231, MCF-7, and SKBR3 (Fig. 3A,B). Using a CCK-8 assay, we found that inhibition of PITX1 significantly decreased the proliferation rate of breast cancer cells (Fig. 3C). Suppression of PITX1 also attenuated the colony-forming potential of MDA-MB-231, MCF-7, and SKBR3 breast cancer cells (Fig. 3D). In addition, knockdown or overexpression of PITX1 markedly decreased or increased, respectively, the migration ability of MDA-MB-231, MCF-7, and SKBR3 breast cancer cells (Fig. 3E,F).

### 3.3 PITX1 Accelerates the Glycolytic Process in Breast Cancer

**Figure 4:**
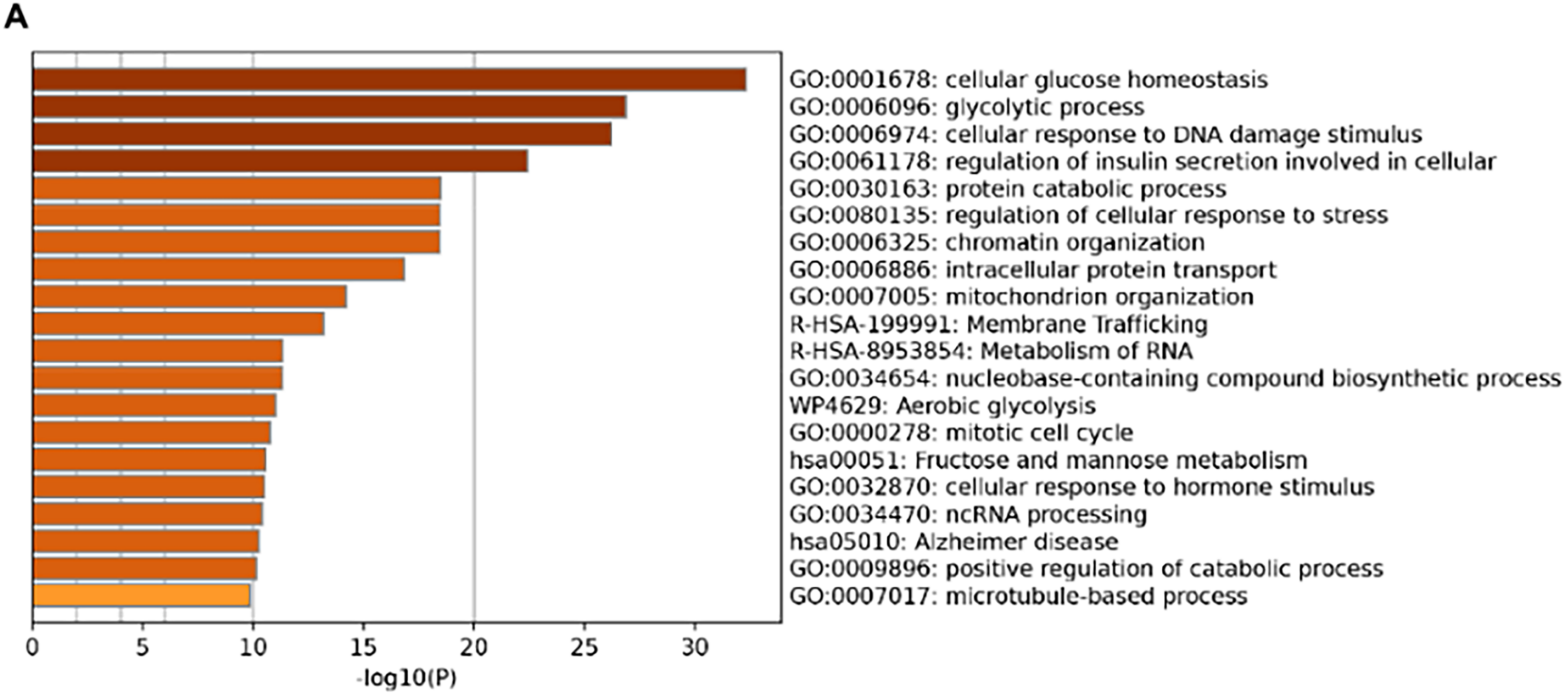

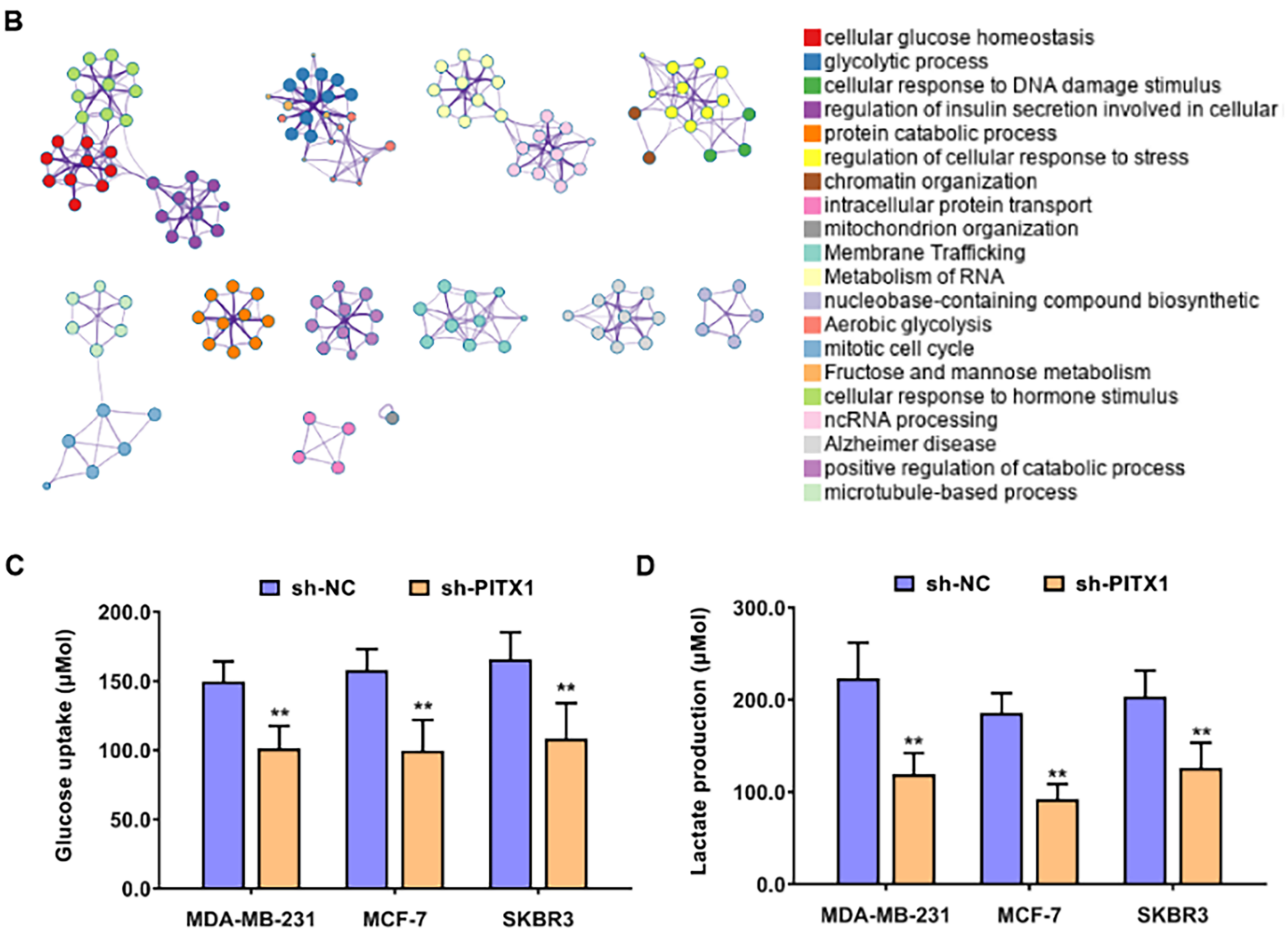
PITX1 accelerates the glycolytic process in breast cancer. (**A**) GO enrichment analysis suggested that the biological function of PITX1 was related to the glycolytic pathway. (**B**) Metascape pathway network analysis revealed that glycolytic pathways are at the center of PITX1 regulatory pathway networks in breast cancer. (**C**) The glucose uptake rate in MDA-MB-231, MCF-7, and SKBR3 breast cancer cells was reduced after inhibition of PITX1. (**D**) The lactate production rate in MDA-MB-231, MCF-7, and SKBR3 breast cancer cells was reduced after inhibition of PITX1. **p < 0.01.

To investigate the biological role of PITX1 in breast cancer cells, we next performed functional enrichment analysis of PITX1. The co-expression genes of PITX1 were extracted and included in the Metascape functional analysis according to the TCGA-BRCA database. We found that the biological function of PITX1 is related to the glycolytic pathway (Fig. 4A). Pathway network analysis showed that the glycolytic pathway is at the center of the PITX1 regulatory pathway in metastatic breast cancer, indicating that PITX1 is likely to mediate distant breast cancer metastasis through the glycolytic pathway (Fig. 4B). Therefore, glucose consumption and lactate production measurements were performed to validate the glycolysis rate after the knockdown of PITX1. Our results showed that PITX1 accelerated glycolysis by increasing glucose uptake and lactate production in MDA-MB-231, MCF-7, and SKBR3 breast cancer cells (Fig. 4C,D).

### 3.4 PITX1 Transcriptionally Activates PFKP Expression to Promote Breast Cancer Metastasis and Accelerate Glycolysis

**Figure 5:**
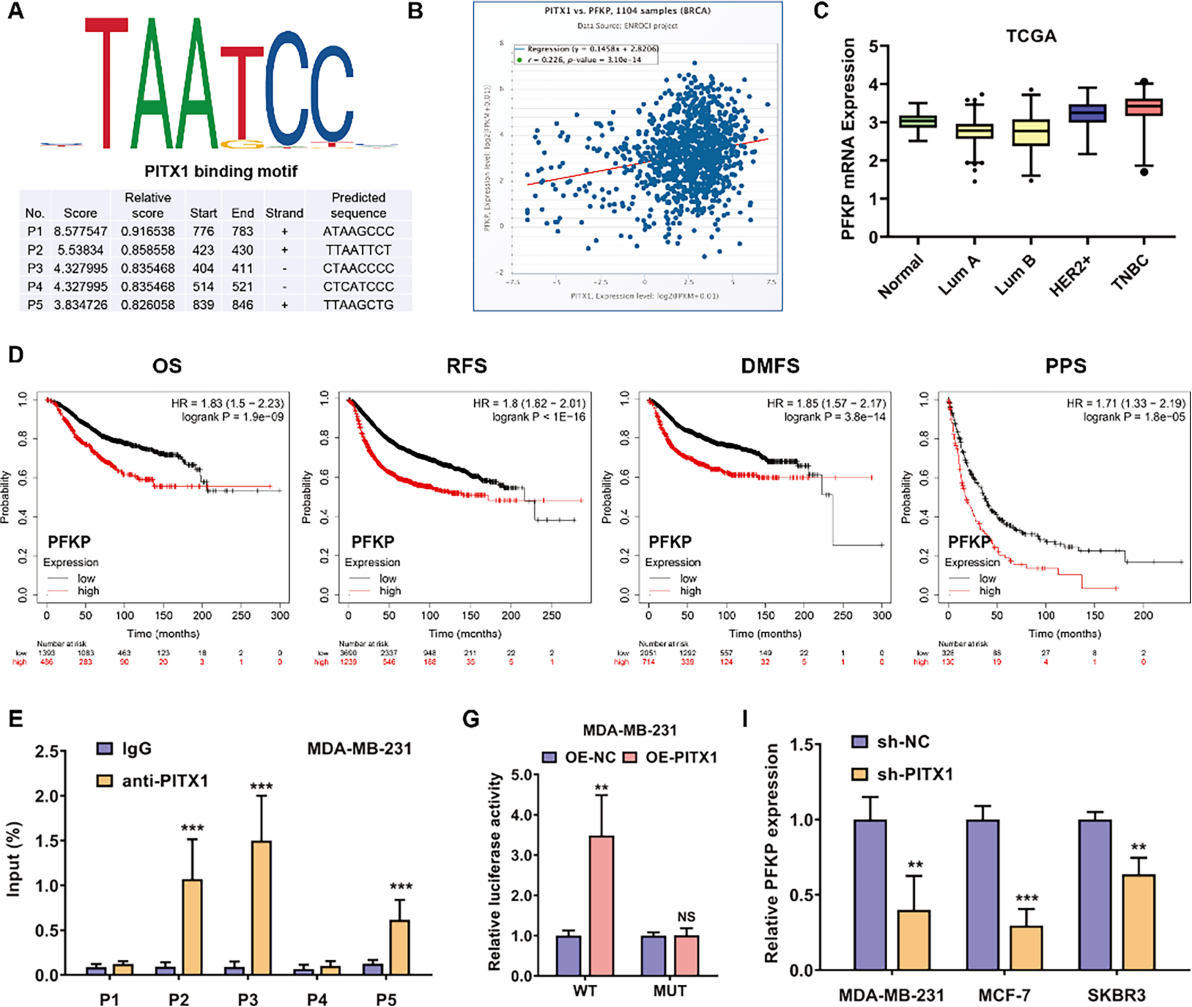

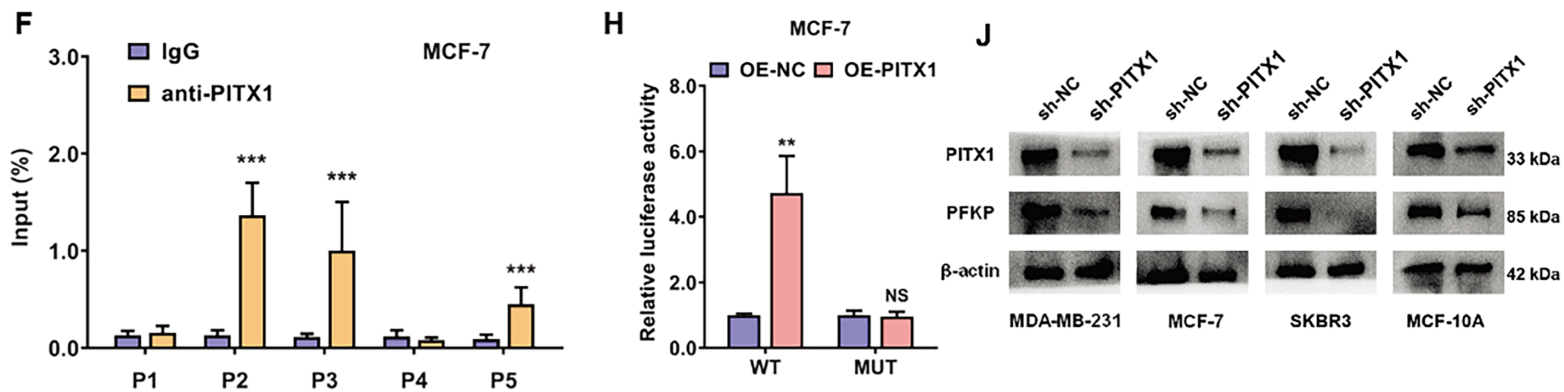
PITX1 transcriptionally activates PFKP expression to promote breast cancer metastasis and accelerate glycolysis. (**A**) The binding motif and sequence of PITX1 were predicted with the JASPAR algorithm. Several binding sites of PITX1 in the promoter DNA sequence of the PFKP locus are displayed. (**B**) The correlation between PITX1 and PFKP expression in breast cancer in the TCGA database. (**C**) The expression profile of PFKP in different molecular subtypes of breast cancer in the TCGA database. (**D**) Higher expression of PFKP was associated with worse overall survival, distant metastasis-free survival, relapse-free survival, and post-progression survival in patients with breast cancer in KM Plotter cohorts. (**E**,**F**) Chromatin immunoprecipitation assays were carried out to evaluate the potential PITX1 binding sites in the PFKP promoter regions. PFKP promoter regions #2, #3, and #5 were the most abundantly enriched by the anti-PITX1 antibody in the MDA-MB-231 and MCF-7 breast cancer cell lines. (**G**,**H**) The luciferase reporter assay revealed that the relative fluorescence intensity was increased after transfection with the PITX1 overexpression plasmid in the wild-type reporter group; however, there was no effect in the mutated reporter group. (**I**) Knockdown of PITX1 decreased the mRNA expression level of PFKP in the MDA-MB-231, MCF-7, and SKBR3 breast cancer cell lines, as revealed by qPCR analysis. (**J**) Inhibition of PITX1 decreased the protein expression level of PFKP in the breast cancer cells (MDA-MB-231, MCF-7, SKBR3) and non-tumorigenic breast epithelial cells (MCF-10A), as revealed by western blot analysis. NS, no significance; **p < 0.01, ***p < 0.001.

**Figure A3:**
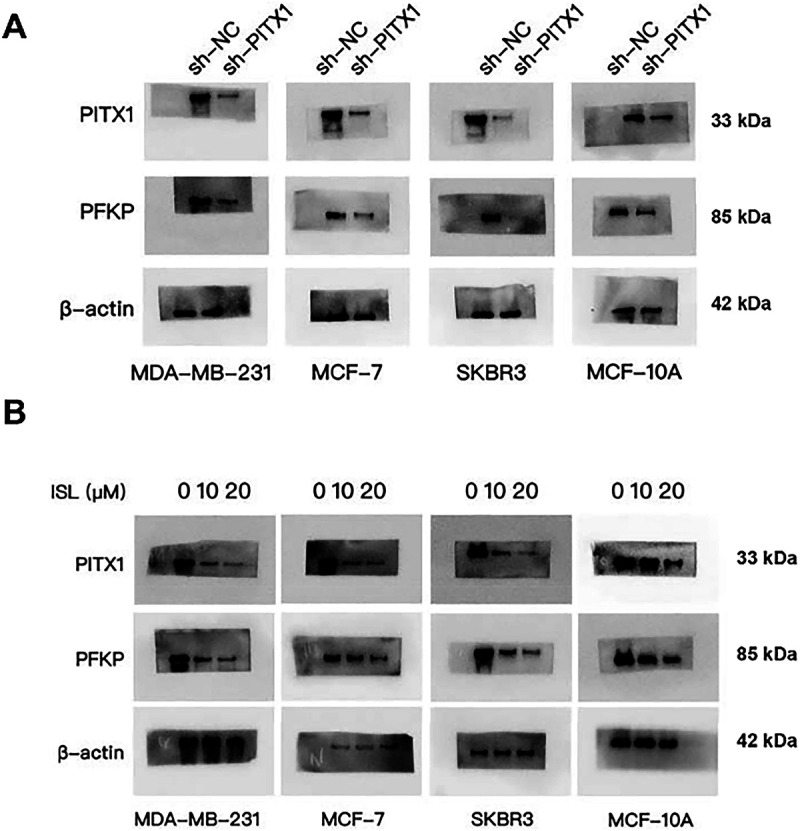
Original uncropped western blot bands used in this study. (**A**) Inhibition of PITX1 decreased the protein expression level of PFKP in the breast cancer cells (MDA-MB-231, MCF-7, SKBR3) and non-tumorigenic breast epithelial cells (MCF-10A), as revealed by western blot analysis. (**B**) Western blot analysis revealed downregulation of PFKP protein levels in breast cancer cells (MDA-MB-231, MCF-7, SKBR3) and non-tumorigenic breast epithelial cells (MCF-10A) after isoliquiritigenin treatment.

We further explored the underlying mechanism by which PITX1 accelerates glycolysis and promotes breast cancer metastasis. As a transcription factor, PITX1 regulates biological processes via transcriptional activation of key downstream genes in cells. To elucidate the potential molecular mechanism by which PITX1 promotes breast cancer progression and cisplatin resistance, we used the JASPAR algorithm to predict its downstream targets. We found several binding sites for PITX1 in the promoter DNA sequence of the PFKP (encoding Phosphohexokinase) locus (Fig. 5A). PFKP is the glycolytic pathway’s core gene, which plays an important role in glycolysis regulation and tumor progression. Several types of cancers, such as clear cell renal cell carcinoma, bladder cancer, breast cancer, and lung cancer, are reprogrammed via PFKP [18–22]. Additionally, there was a strong positive correlation between PITX1 and PFKP expression in breast cancer tissues in the TCGA database (Fig. 5B). Therefore, we hypothesize that PFKP is the downstream target of PITX1 in breast cancer. Next, we explored the expression profile of PFKP in different molecular subtypes of breast cancer (Fig. 5C). Moreover, we found that higher expression of PFKP was associated with worse overall survival, distant metastasis-free survival, relapse-free survival, and post-progression survival in patients with breast cancer in KM Plotter cohorts (Fig. 5D). Then, chromatin immunoprecipitation (ChIP) assays were carried out to evaluate the potential PITX1 binding sites in the PFKP promoter regions. According to the results, PFKP promoter regions #2, #3, and #5 were the most abundantly enriched by the anti-PITX1 antibody in the MDA-MB-231 and MCF-7 breast cancer cell lines (Fig. 5E,F). Furthermore, we assessed the binding sites of PITX1 in the PFKP promoter by a luciferase reporter assay. After transfection with the PITX1 overexpression plasmid, the relative fluorescence intensity was increased in the wild-type reporter group; however, this effect was not observed in the mutated reporter group (Fig. 5G,H). Inhibition of PITX1 decreased the mRNA expression level of PFKP in MDA-MB-231, MCF-7, and SKBR3 breast cancer cells (Fig. 5I). Western blot analysis revealed that the protein expression level of PFKP decreased after the knockdown of PITX1 in both MDA-MB-231 and MCF-7 breast cancer cells (Figs. 5J and A3A).

### 3.5 Isoliquiritigenin Suppresses Breast Cancer Progression via Inhibition of PITX1–PFKP-Mediated Glycolytic Reprogramming

**Figure 6:**
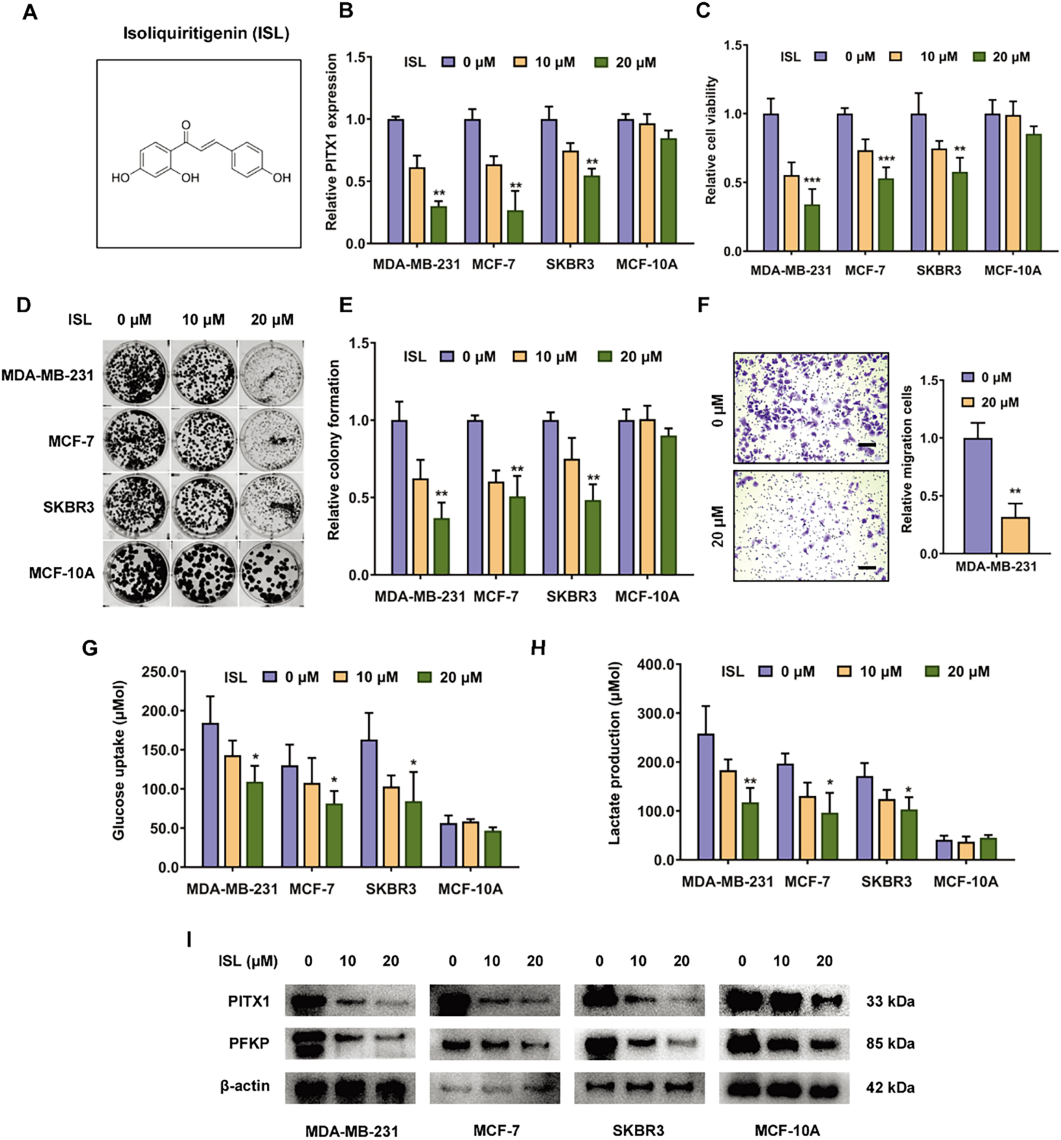
Isoliquiritigenin inhibits the proliferation and metastatic ability of breast cancer cells via targeting PITX1–PFKP-mediated glycolysis reprogramming. (**A**) Chemical structure of isoliquiritigenin. (**B**) Isoliquiritigenin treatment significantly downregulated PITX1 expression in breast cancer cells (MDA-MB-231, MCF-7, SKBR3) and non-tumorigenic breast epithelial cells (MCF-10A). (**C**) CCK-8 assay showing reduced proliferation rates in MDA-MB-231, MCF-7, SKBR3, and MCF-10A cells following isoliquiritigenin treatment. (**D**,**E**) Colony formation assays revealed that isoliquiritigenin significantly inhibited clonogenic potential in MDA-MB-231, MCF-7, SKBR3, and MCF-10A cells. (**F**) Transwell assays demonstrated reduced migratory capacity of MDA-MB-231 cells after isoliquiritigenin-induced PITX1 inhibition. Scale bar: 50 μm. (**G**) Glucose uptake was decreased in MDA-MB-231, MCF-7, SKBR3, and MCF-10A cells following isoliquiritigenin treatment. (**H**) Lactate production was reduced in all three cell lines upon PITX1 inhibition. (**I**) Western blot analysis revealed downregulation of PFKP protein levels in MDA-MB-231, MCF-7, SKBR3, and MCF-10A cells after isoliquiritigenin treatment. *p < 0.05, ** *p* < 0.01, ***p < 0.001.

**Figure 7:**
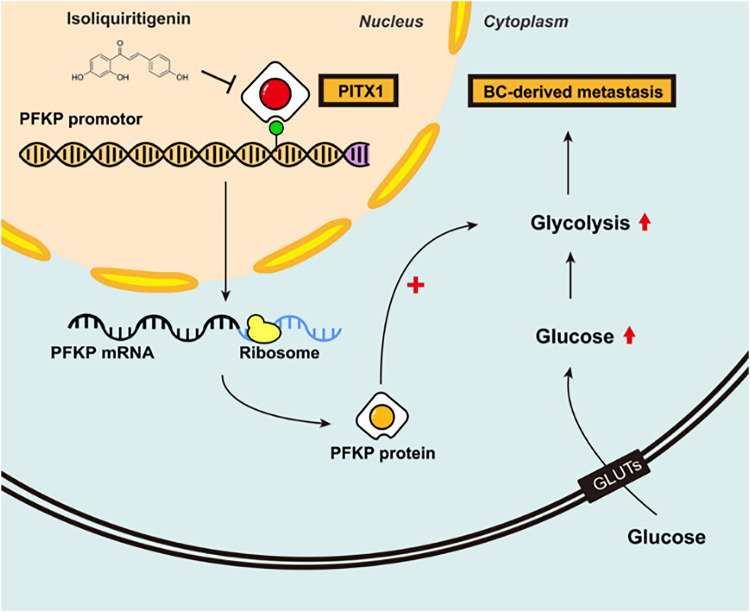
The schematic diagram shows the biological mechanism by which PITX1 transcriptionally activates PFKP expression to facilitate breast cancer growth and metastasis by accelerating glycolysis.

Based on our previous transcriptomic analysis of breast cancer cells treated with isoliquiritigenin, PITX1 was significantly downregulated following treatment, suggesting a potential link between isoliquiritigenin and glycolytic regulation. To explore whether isoliquiritigenin exerts its antitumor effects through the inhibition of PITX1–PFKP-mediated glycolytic reprogramming, we conducted a series of *in vitro* functional assays. First, we confirmed that isoliquiritigenin treatment markedly suppressed PITX1 expression in three breast cancer cell lines with a dose-response effect (MDA-MB-231, MCF-7, and SKBR3) while having a limited effect on the non-tumorigenic breast epithelial cell line MCF-10A (Fig. 6B). Consistently, different doses of isoliquiritigenin significantly inhibited cell proliferation limited effect on the non-tumorigenic breast epithelial cell line MCF-10A, as evidenced by CCK-8 assays (Fig. 6C) and colony formation assays (Fig. 6D,E). Furthermore, cell migration capacity was notably reduced in MDA-MB-231 cells upon isoliquiritigenin treatment, as shown by Transwell assays (Fig. 6F), suggesting an inhibitory effect on metastatic potential. Next, we assessed glycolytic activity following isoliquiritigenin exposure. Treatment significantly reduced glucose uptake (Fig. 6G) and lactate production (Fig. 6H) in all three cell lines, indicating suppression of glycolytic flux. In line with these findings, western blot analysis showed that isoliquiritigenin downregulated PFKP expression, a key glycolytic enzyme transcriptionally regulated by PITX1 (Figs. 6I and A3B). Together, these results suggest that isoliquiritigenin suppresses breast cancer progression by targeting the PITX1–PFKP axis, thereby inhibiting glycolytic reprogramming and reducing both proliferative and metastatic potential of cancer cells. Generally, our results revealed that PITX1 transcriptionally activates PFKP expression to facilitate breast cancer growth and metastasis by accelerating glycolysis (Fig. 7). The findings suggest that targeting PITX1 via isoliquiritigenin could be a promising therapeutic strategy to treat breast cancer in the future.

## 4 Discussion

Metastatic dissemination remains the principal cause of treatment failure and mortality in breast cancer patients [23]. There are fundamental differences in signaling pathways between metastatic and primary cancers [24]. Consequently, defining the regulatory mechanisms that govern breast cancer progression and distant spread continues to be a central focus in oncology research. Multiple biological processes have been implicated in metastatic progression [12]. For instance, Notch pathway activation has been shown to facilitate the metastasis of heterogeneous breast cancer cells through interactions between senescent and adjacent breast cancer cells [25]. In addition, cancer stem-like cells possess enhanced self-renewal capacity and intrinsic resistance to cytotoxic therapies, thereby contributing to metastatic colonization and recurrence [26,27]. Numerous molecular markers have also been associated with metastatic potential in breast cancer [28–30]. Furthermore, reciprocal interactions between tumor cells and components of the tumor microenvironment (TME) have been increasingly recognized as drivers of therapeutic resistance [31–36]. It is still urgent to elucidate the underlying molecular mechanism of progression and develop new therapeutic strategies to overcome metastasis.

In the current study, by analysis of high-throughput RNA sequencing data, PITX1 was identified as an oncogene commonly upregulated in breast cancer. According to immunohistochemical, qPCR, and western blot analyses, PITX1 expression was significantly elevated in breast cancer. In breast cancer patients, higher expression of PITX1 was linked to worse overall survival, distant metastasis-free survival, relapse-free survival, and post-progression survival. We evaluated the role of PITX1 in promoting breast cancer metastasis in both *in vitro* and *in vivo* experiments. The proliferation rate of breast cancer cells was markedly decreased after inhibition of PITX1. Knockdown of PITX1 markedly decreased glycolysis in breast cancer cells.

PITX1 belongs to the homeobox family of transcription factors and exerts its biological effects primarily through promoter binding and transcriptional regulation of downstream genes [37]. Oral mucosa is intrinsically primed for rapid wound repair through baseline activation of wound-associated transcriptional programs, driven in part by SOX2 and PITX1, which regulate epithelial differentiation and inflammation and can reprogram skin keratinocytes to enhance wound healing [38]. Adenocarcinoma patients with high levels of PITX1 expression have poor prognoses, and this effect is associated with DNA methylation [39]. Based on bioinformatics analysis and experimental confirmation, upregulation of the transcription factor PITX1 predicts poor prognosis in clear cell renal cell carcinoma [40]. Cellular metabolic reprogramming (glucose, lipid, amino acid, etc.) is a mechanism by which cells alter their metabolic patterns to meet the energy demands of promoting their proliferation and growth [41,42]. Metabolic reprogramming can increase cellular resistance to external stresses and endow cells with new functions [43]. In our study, we found that PITX1 transcriptionally activates PFKP expression to facilitate breast cancer growth and metastasis by accelerating glycolysis.

PFKP, a key rate-limiting enzyme in glycolysis, plays a central role in metabolic control and has been increasingly linked to tumor aggressiveness [18–20]. For example, R-2-hydroxyglutarate suppresses aerobic glycolysis in leukemia through modulation of the FTO/m6A/PFKP/LDHB signaling axis [20]. Macrophage polarization in the breast cancer microenvironment is mediated by Zeb1-induced glycolytic reprogramming, which PFKP regulates [22]. The PFKP gene is transcriptionally activated by KLFs in breast cancer cells, thereby maintaining a high level of glycolysis. The transcriptional factors can also regulate the immune microenvironment in breast cancer [44]. In addition, it should be noted that homeobox proteins typically do not recognize short DNA motifs of only 4–6 nucleotides in isolation, but instead achieve binding specificity through cooperative interactions with partner proteins that often also engage DNA. Such combinatorial binding is a common mechanism by which homeobox transcription factors regulate gene expression and may contribute to context-dependent transcriptional regulation in this study. Our research also uncovered a new mechanism by which the PFKP gene is transcriptionally activated by PITX1, which promotes breast cancer metastasis by accelerating glycolysis.

Despite the strengths of our study, several limitations should be acknowledged. First, although isoliquiritigenin was shown to suppress the PITX1–PFKP axis, the precise molecular mechanism by which ISL regulates PITX1 remains to be fully elucidated. In particular, it is unclear whether ISL directly binds to PITX1, and if so, which functional domain or structural region of PITX1 mediates this interaction. Further studies involving molecular docking, structural analysis, and binding assays are required to clarify the direct regulatory relationship between ISL and PITX1. Second, while our findings were validated in breast cancer cell lines and supported by bioinformatic analyses of clinical datasets, the translational potential of targeting the PITX1–PFKP axis requires further investigation in clinically relevant preclinical models. Patient-derived xenograft (PDX) and patient-derived organoid (PDO) models would provide more physiologically relevant systems to evaluate therapeutic efficacy and heterogeneity. Future studies incorporating these models are warranted to assess the clinical applicability of ISL or PITX1-targeted strategies in breast cancer.

## 5 Conclusions

In conclusion, this study elucidated the function and mechanism of PITX1 in breast cancer progression and invasion. The findings suggest that targeting PITX1 via isoliquiritigenin could be a promising therapeutic strategy to treat breast cancer in the future.

## Data Availability

The data supporting the findings of this study are available from the following sources: 1. This study utilized publicly available data from The Cancer Genome Atlas (TCGA) database (https://portal.gdc.cancer.gov/). 2. Experimental datasets generated in this study (including qPCR, Western blot, and glycolysis assays) are available from the corresponding author upon reasonable request. 3. Due to patient privacy and ethical considerations, the clinical and RNA sequencing data analyzed in this study are not publicly accessible. However, these data may be shared upon reasonable request to the corresponding authors, subject to approval by the Ethics Committee of the First People’s Hospital of Changde City.
